# Absence of an association of human polyomavirus and papillomavirus infection with lung cancer in China: a nested case–control study

**DOI:** 10.1186/s12885-016-2381-3

**Published:** 2016-06-01

**Authors:** Danny V. Colombara, Lisa E. Manhart, Joseph J. Carter, Stephen E. Hawes, Noel S. Weiss, James P. Hughes, You-Lin Qiao, Philip R. Taylor, Jennifer S. Smith, Denise A. Galloway

**Affiliations:** Department of Epidemiology, School of Public Health, University of Washington, Seattle, WA USA; Fred Hutchinson Cancer Research Center, Seattle, WA USA; Department of Biostatistics, School of Public Health, University of Washington, Seattle, WA USA; Department of Cancer Epidemiology, Cancer Institute, Chinese Academy of Medical Sciences, Beijing, China; Genetic Epidemiology Branch, Division of Cancer Epidemiology and Genetics, National Cancer Institute, Bethesda, USA; Department of Epidemiology, Gillings School of Global Public Health, University of North Carolina, Chapel Hill, NC USA; Institute for Health Metrics and Evaluation, University of Washington, 2301 5th Avenue, Suite 600, Seattle, WA 98121 USA

**Keywords:** Lung cancer, Human polyomavirus, KI polyomavirus, WU polyomavirus, Merkel cell polyomavirus, Human papillomavirus

## Abstract

**Background:**

Studies of human polyomavirus (HPyV) infection and lung cancer are limited and those regarding the association of human papillomavirus (HPV) infection and lung cancer have produced inconsistent results.

**Methods:**

We conducted a nested case–control study to assess the association between incident lung cancer of various histologies and evidence of prior infection with HPyVs and HPVs. We selected serum from 183 cases and 217 frequency matched controls from the Yunnan Tin Miner’s Cohort study, which was designed to identify biomarkers for early detection of lung cancer. Using multiplex liquid bead microarray (LBMA) antibody assays, we tested for antibodies to the VP1 structural protein and small T antigen (ST-Ag) of Merkel cell, KI, and WU HPyVs. We also tested for antibodies against HPV L1 structural proteins (high-risk types 16, 18, 31, 33, 52, and 58 and low-risk types 6 and 11) and E6 and E7 oncoproteins (high risk types 16 and 18). Measures of antibody reactivity were log transformed and analyzed using logistic regression.

**Results:**

We found no association between KIV, WUV, and MCV antibody levels and incident lung cancer (P-corrected for multiple comparisons >0.10 for all trend tests). We also found no association with HPV-16, 18, 31, 33, 52, and 58 seropositivity (P-corrected for multiple comparisons >0.05 for all).

**Conclusions:**

Future studies of infectious etiologies of lung cancer should look beyond HPyVs and HPVs as candidate infectious agents.

**Electronic supplementary material:**

The online version of this article (doi:10.1186/s12885-016-2381-3) contains supplementary material, which is available to authorized users.

## Background

In China, lung cancer is the most commonly diagnosed cancer in males, the second most common in females, and the leading cause of cancer related death for both sexes by a substantial margin [1]. The burden of lung cancer in China is also rising, with disability-adjusted life years per 100,000 increasing by more than 50 % between 1990 (552, 95 % confidence interval (CI): 458–782) and 2010 (843, 95 % CI: 590–990) [2]. Smoking, air pollution (industrial emissions, cooking exhaust, second hand smoke, and residential radon), and genetics are established lung cancer risk factors that explain the majority, but not all, of this burden of disease [3, 4]. However, seven viruses are known to be causally associated with human cancers [5], with their carcinogenic potential often realized only in the presence of environmental mutagens and other cofactors [6]. Due to the lung’s propensity for infection, it is possible that some lung cancers may have an infectious etiology.

The 2008 discovery of Merkel cell carcinoma’s association with Merkel cell polyomavirus (MCV) provided the first evidence that human polyomaviruses (HPyVs) may have carcinogenic potential in humans [7]. Since MCV and other polyomaviruses such as KI (KIV) and WU (WUV), can infect the lower respiratory tract [8–10], their association with lung cancer has been previously examined, but results have been inconclusive. Small MCV studies have reported prevalence estimates of up to nearly 40 % for MCV DNA in lung tumors [11–14]. Less consistency has been observed in studies of the association of KIV and WUV with lung cancer. A small European study reported that KIV DNA was found in 45 % (9/20) of lung tumors but only 5 % (1/20) of control tissue [15]. However, these results were not confirmed by additional studies which examined KIV and or WUV in lung tumors [16–18]. Despite prior infection with human polyomaviruses being nearly ubiquitous [19], prior studies report that high levels of MCV antibodies were associated with Merkel cell carcinoma [20]. However, in the only other seroepidemiologic study, antibodies to MCV, KIV and WUV were not associated with lung cancer in a US population [21]. Nevertheless, given the influence of host genetics on susceptibility to cancer, these associations may differ in different populations.

The human papillomavirus (HPV) family has undisputed carcinogenic potential, with HPV infection playing a causal role in nearly all cervical cancers, a large proportion of other anogenital cancers, and more than a quarter of oropharyngeal cancers [22, 23]. In addition, HPV infections are involved in the development of respiratory papillomas [24], which occasionally exhibit malignant transformation [25]. Based on this evidence, there have been numerous studies of the association between HPV infection and lung cancer. In 2009, a meta-analysis and a systematic review independently concluded that the evidence for an association remained inconclusive, but stronger associations were observed in East Asia [26, 27]. More recently, a 2015 meta-analysis concluded that HPV infection is associated with increased risk for lung cancer [28]. However, only nine studies were included in the analysis, with all of the studies having tested lung tissue for current evidence of infection as opposed to longitudinally collected sera that could be used to assess prior infection. In addition, the degree of heterogeneity reported was not inconsequential and the meta-analysis used only crude data, which was unadjusted for potential confounders such as age and smoking status [29]. Finally, as the authors noted, there were some indications of publication bias. Therefore, HPV’s association with lung cancer remains an open question.

## Methods

### Study population

Between 1992 and 1998, 9,295 eligible Yunnan Tin Corporation (YTC) employees were enrolled in a cohort study of early markers of lung cancer in Yunnan, China [30]. Participants were current or retired YTC workers, at least 40 years of age, with a history of at least 10 years underground and/or smelting experience, and no previous malignancies (except non-melanoma skin cancer).

### Data and specimen collection

Participants completed an interviewer-administered questionnaire regarding demographics, lung cancer symptoms, eating habits, and medical, smoking and occupational history. They also received at least one yearly screening exam consisting of sputum cytology, chest x-rays, and a physical exam provided by the YTC General Worker’s Hospital. All positive, and 2 % of negative, cytology slides were re-read for diagnosis and adequacy of preparation by YTC and a Johns Hopkins University pathologist. Two radiologists read each chest x-ray, with differences resolved by a third reader. In addition, yearly sputum specimens and a one-time sample of urine, 10 mL of whole blood, and toenail clippings were collected and preserved for future studies. Plasma was separated from whole blood and stored at −70 °C. Follow-up activities ceased on December 31, 2001.

Participants provided signed informed consent and the institutional review boards (IRBs) of the National Cancer Institute (NCI) and the YTC approved the original cohort study. The IRBs of the University of Washington, the Fred Hutchinson Cancer Research Center, the Cancer Institute of the Chinese Academy of Medical Sciences, and the NCI approved this analysis.

### Case definition

Cases (n = 183) are defined as individuals with incident lung cancer of any histology (small cell carcinoma, squamous cell carcinoma, adenocarcinoma, and other). Incident lung cancer was determined in one of four ways: 1) detection during annual screenings utilizing chest x-rays and sputum cytology, followed-up by confirmatory diagnosis at the YTC General Worker’s Hospital, 2) presentation to and diagnosis at the YTC General Worker’s Hospital among those with symptoms, 3) searching the YTC cancer registry system which contains data from local hospitals, or 4) searching hospital based vital records for deceased cohort members and identifying the cause of death. Controls (n = 217) were frequency matched on age and the number of freeze-thaw cycles. Since only two of the lung cancer cases occurred in women, this study was limited to men.

### Exposure assessment

We used a Bio-Plex 200 instrument (Bio-rad Laboratories) to perform multiplex liquid bead microarray (LBMA) antibody assays following standard procedures [20, 31, 32]. We obtained and analyzed the median fluorescent intensity (MFI), a surrogate for antibody titer. We tested sera for antibodies against the VP1 structural protein and small T antigen (ST-Ag) oncoprotein of Merkel cell, KI, and WU HPyVs. In addition, we tested for antibodies against HPV L1 structural proteins (high-risk types 16, 18, 31, 33, 52, and 58 and low-risk types 6 and 11) and E6 and E7 oncoproteins (high risk types 16 and 18 only). Glutathione S-transferase (GST) fusion proteins and BK virus antigens served as negative and positive controls, respectively. All fusion proteins had an eleven amino acid epitope “Tag” added to the C-terminus to assess expression levels [20]. Between 89.3 % and 95 % of sera were seropositive for BK VP1, depending on whether we used a threshold of 400 or 200 MFI, respectively. Sera were incubated at a final concentration of 1:100.

### Statistical methods

Since HPyV infection was expected to be common, we compared the range of MFI between cases and controls rather than dichotomizing specimens as seropositive or seronegative. We assessed the association between HPyV infection and lung cancer using MFI quartiles as the independent variable in logistic regression analyses. In contrast, we dichotomized HPV MFI in order to increase comparability of our HPV analysis with prior LBMA based studies. We defined HPV seropositivity as >400 MFI (>5.99 log transformed MFI (lnMFI)) [31, 33, 34] in our primary analysis and >200 MFI (>5.30 lnMFI) [34, 35] in a sensitivity analysis. We also assessed the association between each viral antibody and incident lung cancer using logistic regression trend tests with continuous lnMFI as the independent variable. MFI were log transformed and all logistic regression analyses were adjusted for matching variables.

Since a total of 18 antibodies were assessed for an association with incident lung cancer, we created exposure categories to account for multiple comparisons. We created three categories of HPyV exposure: MCV (VP1 and ST-Ag), KIV (VP1 and ST-Ag), and WUV (VP1 and ST-Ag). Similarly, four categories of HPV exposure were created: low-risk HPV (6, 11 L1), HPV-16 (E6, E7, and L1), HPV-18 (E6, E7, and L1), and other high-risk HPV (31, 33, 52, 58 L1). We corrected our *P*-values for multiple comparisons by using permutation tests with 10,000 permutations to establish a null distribution of the most significant exposure across the multiple exposures in each exposure category [36]. The proportion of the time that the empirical test statistic was less than or equal to the test statistic calculated using permuted datasets was defined as the corrected *P*-value.

We assessed effect modification by smoking history (linear pack-years), radon exposure (Working Level Month (WLM)), and arsenic exposure (Index of Arsenic Exposure Months (IAEM)) using likelihood ratio tests. If radon, and arsenic exposure were not effect modifiers, they were considered potential confounders, along with smoking history and a family history of lung cancer (yes, no). If inclusion of a candidate confounder in the regression models changed the odds ratio (OR) of interest by < 10 %, that candidate was not included in the final model.

In exploratory analyses, boxplots were used to assess the association of antigen-specific MFI with individual histologic types.

All analyses used two-sided statistical tests and were performed with Stata/IC 13.1 (StataCorp LP, College Station, TX).

## Results

The age distribution was similar between cases and controls, but the controls were better educated (P = 0.04) (Table 1). Cases were more likely to have ever smoked tobacco (96.7 % vs. 90.8 %, P = 0.02), but had similar overall levels of tobacco, arsenic, and radon exposure. Nearly two-fifths of the cases had squamous cell carcinoma (39.3 %), 16.9 % had adenocarcinoma, 13.7 % had small cell carcinoma, 10.4 % had a mixed histology, and 19.7 % had other histologies or the histology was not obtained.

**Table 1:** Characteristics of selected lung cancer cases and frequency matched controls: Yunnan, China 1992-1998

Characteristic	Cases (n = 183)		Controls (n = 217)		P
	n	%	n	%	
Age					0.914
40–49	3	(1.6)	4	(1.8)	
50–59	58	(31.7)	71	(32.7)	
60–69	104	(56.8)	125	(57.6)	
70–79	18	(9.8)	17	(7.8)	
Education level^a^					0.043
None	82	(44.8)	87	(40.1)	
K–5	89	(48.6)	98	(45.2)	
6–8	9	(4.9)	15	(6.9)	
9–12	2	(1.1)	5	(2.3)	
College	1	(0.5)	12	(5.5)	
Body Mass Index (BMI) (kg/m^2^)					0.230
Underweight (<18.5)	18	(9.8)	36	(16.6)	
Normal (18.5–24.99)	138	(75.4)	150	(69.1)	
Overweight (25–29.99)	24	(13.1)	29	(13.4)	
Obese (≥30)	3	(1.6)	2	(0.9)	
Ever smoked tobacco^b^	177	(96.7)	197	(90.8)	0.016
Pack-years, median (IQR^c^)	36	(24–54)	32	(19–49)	0.132
Arsenic^d^, median (IQR^c^)	10745	(6695–15347)	9420	(4888–16802)	0.057
Radon^e^, median (IQR^c^)	498	(277–783)	417	(171–681)	0.132
Family history of lung cancer^f^	3	(1.6)	6	(2.8)	0.449
Any prior lung disease^g^	88	(48.1)	98	(45.2)	0.559
Asthma or hay fever	25	(13.7)	24	(11.1)	0.429
Tuberculosis (self-report)	8	(4.4)	14	(6.5)	0.363
Chronic bronchitis	72	(39.3)	78	(35.9)	0.484
Silicosis	20	(10.9)	18	(8.3)	0.371
Lung cancer histology					NA
Squamous cell carcinoma	72	(39.3)	0	(−)	
Adenocarcinoma	31	(16.9)	0	(−)	
Small cell carcinoma	25	(13.7)	0	(−)	
Mixed	19	(10.4)	0	(−)	
Other	2	(1.1)	0	(−)	
Not obtained	34	(18.6)	0	(−)	
Lung cancer site					NA
Main bronchus	15	(8.2)	0	(−)	
Upper lobe bronchus or lung	76	(41.5)	0	(−)	
Middle lobe lung	19	(10.4)	0	(−)	
Lower lobe bronchus or lung	60	(32.8)	0	(−)	
Other parts of bronchus or lung	3	(1.6)	0	(−)	
Bronchus and lung NOS	10	(5.5)	0	(−)	

^a^ Highest educational level started

^b^ Having ever smoked tobacco was defined as having smoked cigarettes, pipes, or water pipes for 6 months or longer, or providing an age for beginning or quitting smoking, or providing a non-zero measure of tobacco smoked daily

^c^ IQR, interquartile range

^d^ Measured in iaem, index of arsenic exposure months, a time weighted arsenic exposure measurement (mg/m^3^ x months)

^e^ Measured in wlm, working level month

^f^ Family history of lung cancer was defined as having any immediate family member (parents, siblings, children, or spouse) who received a doctor’s diagnosis of lung cancer

^g^ Any prior lung disease was defined as a prior diagnosis of asthma or hay fever, tuberculosis, chronic bronchitis, or silicosis.

The distribution of antigen specific antibodies was similar among lung cancer cases and controls (Table 2). The maximum difference in the mean lnMFI between cases and controls was 0.3 for HPV 16 E7 and HPV 11 L1 antibodies.

**Table 2:** The distribution of antigen specific antibodies^a^ among lung cancer cases and controls

	Cases		Controls		
	(n = 183)		(n = 217)		
Antibody	Mean	SD	Mean	SD	Difference
HPyV					
MCV^b^ VP1^c^	8.4	2.5	8.4	2.5	0
MCV^b^ ST-Ag^d^	1.1	1.8	1.1	2	0
KIV^e^ VP1 ^c^	9	1.9	8.9	1.5	0.1
KIV^e^ ST-Ag^d^	1.9	1.9	1.9	1.9	0
WUV^f^ VP1 ^c^	9.2	1	9.1	.8	0.1
WUV^f^ ST-Ag^d^	1.7	1.8	1.6	1.7	0.1
HPV^g^ 16					
E6	.4	1.2	.2	1	0.2
E7	1.7	2.6	1.4	2.3	0.3
L1	1.3	2.1	1.1	1.9	0.2
HPV^g^ 18					
E6	1.6	2	1.5	1.9	0.1
E7	.6	1.6	.5	1.4	0.1
L1	2.1	2	2.1	1.9	0
Other high-risk HPV^g^					
31 L1	2.6	2.3	2.6	2.5	0
33 L1	1.8	2	1.7	1.9	0.1
52 L1	5	2	5	1.9	0
58 L1	3.5	2.4	3.4	2.3	0.1
Low-risk HPV^g^					
6 L1	5.1	2.7	4.9	2.6	0.2
11 L1	4	2.4	3.7	2.4	0.3

^a^ Measured in units of natural log transformed median fluorescence intensity (lnMFI). The “Mean” is the arithmetic mean, “SD” is the standard deviation, and “Difference” is equal to the mean of the cases minus the mean of the controls

^b^ MCV = Merkel cell polyomavirus

_c_ VP1 = the primary structural protein of human polyomaviruses

_d_ ST-Ag = the small T-antigen of human polyomaviruses

^e^ KIV = KI polyomavirus

^f^ WUV = WU polyomavirus

^g^ HPV = Human papillomavirus

In multivariate analyses there was little evidence for confounding so the models were adjusted only for matching variables in the main analysis. The results of the regression analysis comparing the highest to the lowest quartile of MCV antibodies with respect to incident lung cancer found no appreciable association for either VP1 (aOR = 0.90, 95 % CI: 0.37-2.17) or ST-Ag (aOR = 0.85, 95 % CI: 0.48-1.48) (Table 3). Compared to men with the lowest levels of KIV antibodies, those with the highest quartile of VP1 (aOR = 1.44, 95 % CI: 0.82-2.52) and ST-Ag (aOR = 1.13, 95 % CI: 0.65-1.98) did not face a significantly increased risk of lung cancer. Those with the highest quartile of WUV VP1 (aOR = 1.47, 95 % CI: 0.84-2.58) and WUV ST-Ag (aOR = 1.02, 95 % CI: 0.58-1.78) antibodies also showed no evidence of increased risk. Linear trend tests of these associations confirmed the lack of association (P > 0.10 for all HPyV antibodies). A sensitivity analysis that included adjustment for education and ever smoking, both of which were associated with case status, did not differ substantially from the main analysis (<10 % change in the odds ratio) (Additional file 1: Table S1).

**Table 3:** Association between antigen specific human polyomavirus (HPyV) antibody levels and incident lung cancer, adjusted for matching variables

	Mean			Trend Test^d^	
Antibody quartile	lnMFI^a^	aOR (95%CI^b^)	*P* ^c^	OR (95%CI^b^)	*P* ^c^
MCV^e^ VP1^f^				1.00 (0.92–1.09)	0.951
1	5.03	Referent			
2	8.50	0.67 (0.38–1.17)	0.163		
3	9.74	0.80 (0.49–1.31)	0.372		
4	12.57	0.90 (0.37–2.17)	0.810		
MCV^e^ ST-Ag^g^				0.97 (0.88–1.08)	0.623
1	0.02	Referent			
2	0.05	1.01 (0.58–1.76)	0.985		
3	0.25	0.70 (0.40–1.22)	0.209		
4	4.12	0.85 (0.48–1.48)	0.553		
KIV^h^ VP1^g^				1.04 (0.92–1.17)	0.532
1	6.92	Referent			
2	8.65	1.07 (0.61–1.88)	0.821		
3	9.32	1.64 (0.94–2.89)	0.089		
4	10.81	1.44 (0.82–2.52)	0.206		
KIV^h^ ST-Ag^g^				0.99 (0.90–1.10)	0.878
1	0.02	Referent			
2	0.22	1 .00 (0.58–1.75)	0.991		
3	3.00	0.72 (0.41–1.27)	0.260		
4	4.43	1.13 (0.65–1.98)	0.652		
WUV^i^ VP1^g^				1.20 (0.96–1.51)	0.112
1	8.16	Referent			
2	8.86	1.34 (0.77–2.36)	0.305		
3	9.34	1.15 (0.65–2.01)	0.643		
4	10.13	1.47 (0.84–2.58)	0.182		
WUV^i^ ST-Ag^g^				1.02 (0.91–1.14)	0.734
1	0.02	Referent			
2	0.07	0.95 (0.55–1.67)	0.871		
3	2.40	0.83 (0.47–1.45)	0.522		
4	4.04	1.02 (0.58–1.78)	0.951		

^a^ lnMFI = natural log transformed median fluorescence intensity

^b^ Nominal (uncorrected) 95 % confidence intervals

^c^ *P*-values are corrected for multiple comparisons using permutation tests

^d^ The trend tests estimate the odds ratio for a one unit increase in natural log transformed MFI, adjusted for matched variables

^e^ MCV = Merkel cell polyomavirus

^f^ VP1 = the primary structural protein of human polyomaviruses

_g_ ST-Ag = the small T-antigen of human polyomaviruses

^h^ KIV = KI polyomavirus

^i^ WUV = WU polyomavirus

HPV 16 L1 (aOR = 1.17, 95 % CI: 0.43-3.21) seropositivity was not associated with increased lung cancer risk (Table 4). HPV 18 L1 seropositivity also showed no evidence of increased risk (aOR = 0.29, 95 % CI: 0.03-2.66). Seropositivity to other high-risk HPV types and low-risk HPV types also showed no relationship with lung cancer incidence (adjusted P > 0.05 for all). The aOR for linear trend tests of HPV lnMFI in association with incident lung cancer ranged from 1.00 (95 % CI: 0.90-1.11) for HPV 52 L1 to 1.17 (95 % CI: 0.97-1.43) for HPV16 E6. The sensitivity analysis using a threshold of 5.30 lnMFI also showed no evidence of an association (P > 0.05 for all) (Additional file 2: Table S2). Additional sensitivity analyses that included adjustment for education and ever smoking did not differ substantially from the main analyses with threshold at 5.99 lnMFI or the alternative threshold of 5.30 lnMFI (<10 % change in the odds ratio) (Additional file 3: Table S3 and Additional file 4: Table S4 respectively).

**Table 4:** Association between human papillomavirus (HPV) seropositivity^a^ and incident lung cancer, adjusted for matching variables

Antibody	Cases (n = 200)	Controls (n = 200)			Trend Test^b^	
	%	%	OR (95 % CI^c^)	*P* ^d^	OR (95 % CI^c^)	*P* ^d^
HPV 16						
E6	0.5	1.4	0.39 (0.04–3.79)	0.462	1.17 (0.97–1.43)	0.097
E7	9.3	7.8	1.22 (0.60–2.47)	0.603	1.06 (0.97–1.15)	0.182
L1	4.4	3.7	1.17 (0.43–3.21)	0.746	1.04 (0.95–1.15)	0.374
HPV 18						
E6	1.6	1.4	1.18 (0.23–5.93)	0.877	1.02 (0.93–1.13)	0.632
E7	1.6	1.4	1.18 (0.23–5.93)	0.877	1.06 (0.93–1.21)	0.404
L1	0.5	1.8	0.29 (0.03–2.66)	0.229	1.00 (0.91–1.11)	0.948
Other high-risk HPV						
31 L1	6.6	8.8	0.72 (0.34–1.53)	0.402	1.00 (0.92–1.09)	0.988
33 L1	0.5	0.5	1.23 (0.08–20.03)	0.887	1.03 (0.93–1.15)	0.537
52 L1	34.4	33.6	1.04 (0.68–1.57)	0.883	1.00 (0.90–1.11)	0.989
58 L1	13.7	12.0	1.17 (0.65–2.10)	0.611	1.03 (0.94–1.12)	0.543
Low-risk HPV						
6 L1	45.4	38.2	1.34 (0.89–2.00)	0.167	1.03 (0.95–1.11)	0.503
11 L1	20.2	13.8	1.58 (0.93–2.68)	0.097	1.05 (0.97–1.14)	0.229

^a^ Seropositivity defined as >400 MFI (median fluorescence intensity)

^b^ The trend tests estimate the odds ratio for a one unit increase in natural log transformed MFI, adjusted for matched variables

^c^ Nominal (uncorrected) 95 % confidence intervals

^d^ *P*-values are corrected for multiple comparisons using permutation tests

**Fig 1:**
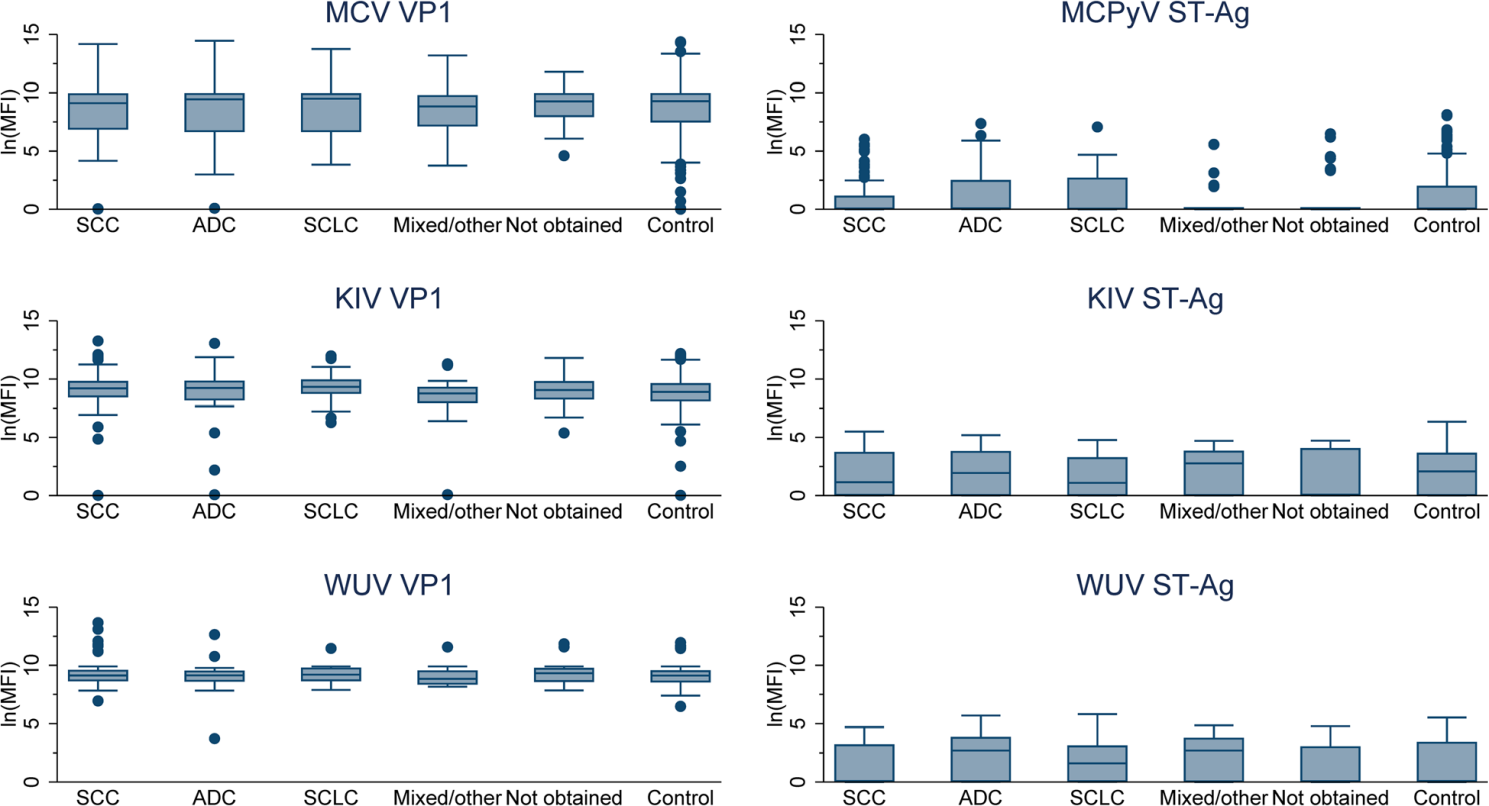
Boxplots of human polyomavirus (HPyV) antigen specific antibody distributions, by lung cancer histology type. *The shaded box represents the inter-quartile range (IQR), the horizontal line within the box represents the median, the vertical lines extend to 1.5 times the IQR, and dots represent outliers. **Abbreviations: ln(MFI), natural log median fluorescence intensity; SCC, squamous cell carcinoma; ADC, adenocarcinoma; SCLC, small cell lung cancer

**Fig 2:**
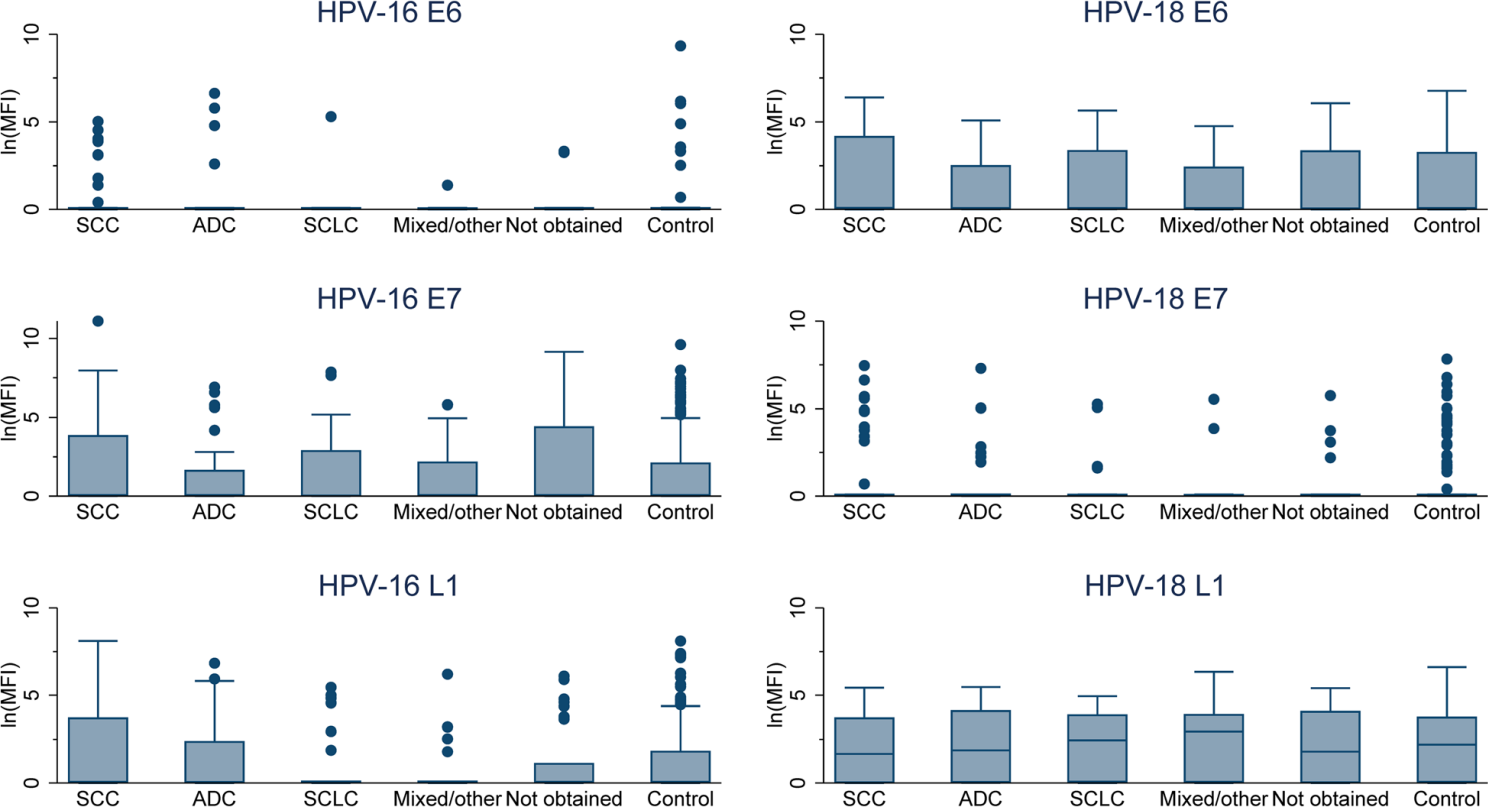
Boxplots of human papillomavirus (HPV) 16 and 18 antigen specific antibody distributions, by lung cancer histology type. *The shaded box represents the inter-quartile range (IQR), the horizontal line within the box represents the median, the vertical lines extend to 1.5 times the IQR, and dots represent outliers. **Abbreviations: ln(MFI), natural log median fluorescence intensity; SCC, squamous cell carcinoma; ADC, adenocarcinoma; SCLC, small cell lung cancer

**Fig 3:**
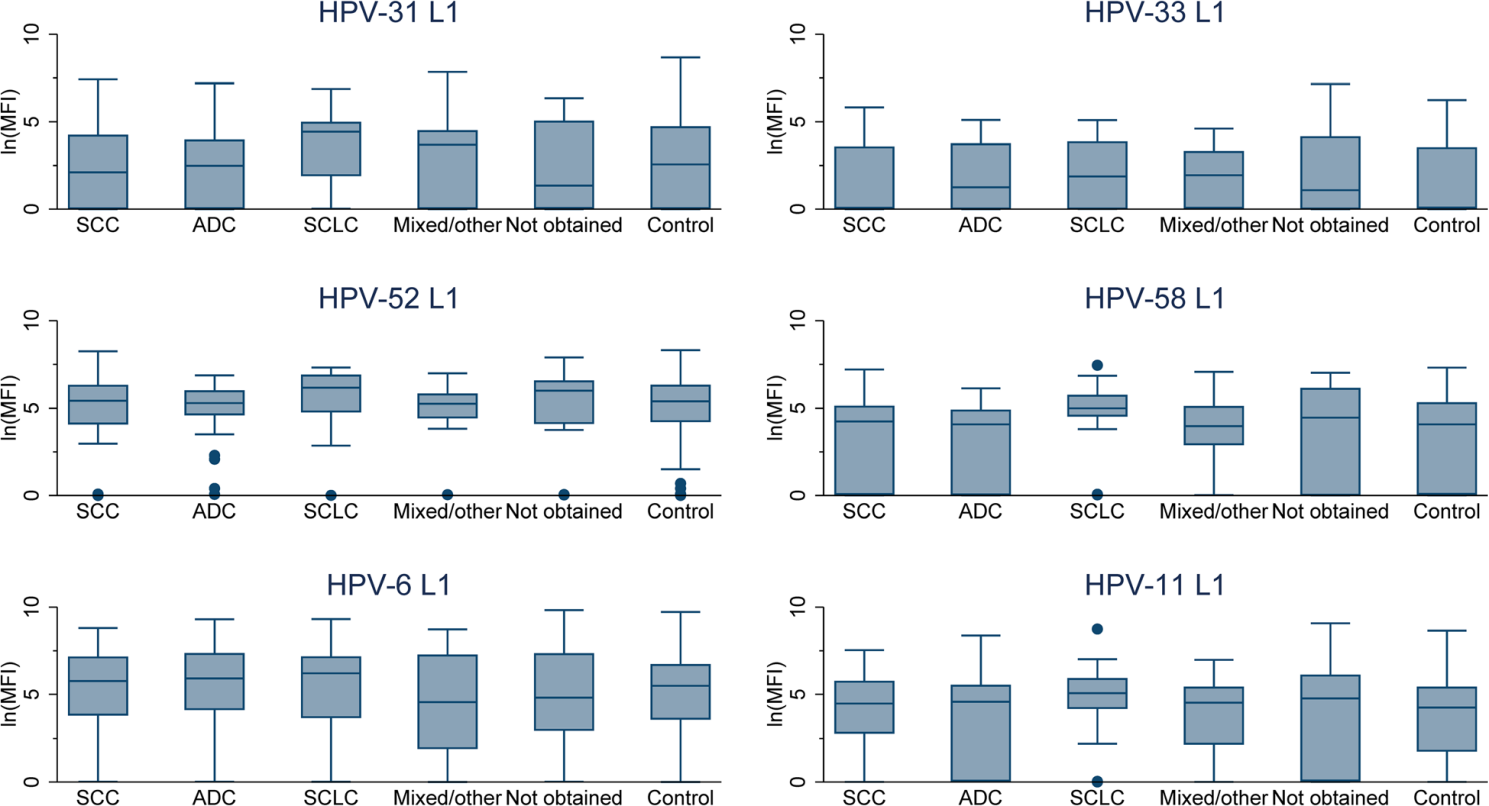
Boxplots of high-risk (31, 33, 52 and 58) and low-risk (6 and 11) human papillomavirus (HPV) antigen specific antibody distributions, by lung cancer histology type. *The shaded box represents the inter-quartile range (IQR), the horizontal line within the box represents the median, the vertical lines extend to 1.5 times the IQR, and dots represent outliers. **Abbreviations: ln(MFI), natural log median fluorescence intensity; SCC, squamous cell carcinoma; ADC, adenocarcinoma; SCLC, small cell lung cancer

Exploratory boxplots showed no evidence of an association between antigen-specific antibodies and any specific lung cancer histology (Figs. 1, 2 and 3).

## Discussion

In this seroepidemiologic study of the association of HPyV and HPV and incident lung cancer in Asia, multivariable regression analyses of pooled histology types and visual exploration of boxplots stratified by histology type demonstrated no evidence of an association between the levels of these viral antibodies and lung cancer. These results are consistent with our previous seroepidemiologic study in an American population, which also found no association between HPyV antibody levels and lung cancer [21].

Only one small European study using nucleic acid amplification testing (NAAT) to detect viral DNA found a strong association between lung cancer of undescribed histology and KIV [15]. Subsequent NAAT based studies of KIV and small cell carcinoma and large cell neuroendocrine carcinoma [16], adenocarcinoma [17], and small cell neuroendocrine carcinoma [18] all failed to confirm these positive findings. Among the studies of MCV and WUV in association with lung cancer, the majority did not have a true comparison group, which limits the ability to determine possible associations [11, 13, 14, 16–18]. One study that assessed tumors along with adjacent benign tissue reported finding MCV DNA in 5 of 30 cases as compared to 2 of 21 controls [12], which provides a non-statistically significant OR of 1.9.

In contrast to meta-analyses which reported potential associations between lung cancer and HPV in East Asia [27, 28, 37], we found no evidence of an association with seropositivity to eight different HPV types. To date, more than 100 HPV / lung cancer association studies have been conducted [37] and wide ranging differences in reported associations among East Asian countries have been noted [26]. Since our sero-assay was less sensitive than NAATs, it remains unclear whether previous positive associations were due to contamination [38, 39] or whether negative associations were due to differences in selected primers [26, 27] and the use of formalin-fixed paraffin embedded specimens, which may hinder attempts to amplify longer DNA segments [27, 40].

After completing the study and analyzing the data, we found that ever smoking status and level of education were associated with case/control status. Both factors could have potentially confounded the relationship between viral infection and lung cancer. While we were unable perform the analysis anew with matching on smoking history and education, we were able to assess confounding by including these factors as adjustment variables in sensitivity analyses. Since inclusion of these variables in the models did not substantially change the interpretation of our results, we reported the analysis adjusted for matching variables as our primary findings.

The primary limitation of this study is that all cohort members were at high risk for lung cancer due to occupational exposures. In the absence of interactive effects, these potent risk factors may obscure weaker contributions to lung cancer risk that might be observed in a broader population sample. This was by design, since we originally hypothesized that both HPVs and HPyVs might have an interactive effect with known carcinogens to contribute to lung cancer risk [41–43]. In addition, the study was underpowered for the examination of these associations by histologic type. However, some HPV types have been reported to be strongly associated with squamous cell carcinoma [28] and Merkel cell carcinoma is histologically similar to small cell carcinoma [44], so there may not have been an ideal distribution of histologic types for this study. The lack of specificity regarding the location of infection is also a limitation of our design. However, based on the limited number of seropositive samples, this is unlikely to have been important in this analysis. We also acknowledge that serology is not a gold standard for the evaluation of carcinogenic viruses associated with solid cancers [45]. Nevertheless, seroepidemiologic methods allow for ready selection of appropriate controls and are uniquely positioned to detect evidence for hit-and-run viral oncogenesis [46]. Finally, as mentioned above, another limitation is the potential lack of assay sensitivity compared to NAATs. The primary strengths of this study are the reduced susceptibility to contamination compared to NAATs and the temporal element that would have better informed causal inference if we did observe an association.

## Conclusions

In summary, we found no association between KIV, WUV, and MCV antibody levels or HPV-16, 18, 31, 33, 52, and 58 seropositivity and incident lung cancer in a high-risk male Asian cohort. Future studies of infectious etiologies of lung cancer should look beyond HPyVs and HPVs as candidate infectious agents.

## Abbreviations

CI, confidence interval; HPV, human papillomavirus; HPyV, human polyomavirus; KIV, KI polyomavirus; LBMA, liquid bead microarray; MFI, median fluorescent intensity; NAAT, nucleic acid amplification test; NCI, National Cancer Institute; ST-Ag, small T antigen; WUV, WU polyomavirus; YTC, Yunnan Tin Corporation

## Additional files

Additional file 1:
**Table S1.** Association between antigen specific human polyomavirus (HPyV) antibody levels and incident lung cancer, adjusted for matching variables, ever smoking, and years of education. (DOCX 15 kb) Additional file 2:
**Table S2.** Association between human papillomavirus (HPV) seropositivity^a^ and incident lung cancer, adjusted for matching variables. (DOCX 26 kb) Additional file 3:
**Table S3.** Association between human papillomavirus (HPV) seropositivity^a^ and incident lung cancer, adjusted for matching variables, ever smoking, and years of education. (DOCX 14 kb) Additional file 4:
**Table S4.** Association between human papillomavirus (HPV) seropositivity^a^ (alternate threshold) and incident lung cancer, adjusted for matching variables, ever smoking, and years of education. (DOCX 26 kb)
