# Supplementary material for: Absence of an association of human polyomavirus and papillomavirus infection with lung cancer in China: a nested case–control study
Source: BMC Cancer. 2016 Jun 1;16:342. doi: 10.1186/s12885-016-2381-3 (PMC4888628; doi:10.1186/s12885-016-2381-3)
Supplement: Additional file 1: — Table S1. Association between antigen specific human polyomavirus (HPyV) antibody levels and incident lung cancer, adjusted for matching variables, ever smoking, and years of education. (DOCX 15 kb) [file 12885_2016_2381_MOESM1_ESM.docx]

**Supplemental Table 1.** Association between antigen specific human polyomavirus (HPyV) antibody levels and incident lung cancer, adjusted for matching variables, ever smoking, and years of education.

|  | | | **Mean** |  |  | **Trend Test**^d^ | |
| --- | --- | --- | --- | --- | --- | --- | --- |
| **Antibody quartile** | | | **lnMFI**^a^ | **aOR (95%CI**^b^**)** | ***P***^c^ | **OR (95%CI**^b^**)** | ***P***^c^ |
| **MCV**^e^ **VP1**^f^ | |  |  |  |  | 1 (0.92-1.09) | 0.988 |
|  | 1 | | 5.03 | Referent |  |  |  |
|  | 2 | | 8.50 | 0.71 (0.40-1.25) | 0.237 |  |  |
|  | 3 | | 9.74 | 0.79 (0.48-1.31) | 0.363 |  |  |
|  | 4 | | 12.57 | 0.92 (0.38-2.25) | 0.860 |  |  |
| **MCV**^e^ **ST-Ag**^g^ | |  |  |  |  | 0.98 (0.88-1.09) | 0.704 |
|  | 1 | | 0.02 | Referent |  |  |  |
|  | 2 | | 0.05 | 1.03 (0.58-1.81) | 0.920 |  |  |
|  | 3 | | 0.25 | 0.67 (0.38-1.18) | 0.174 |  |  |
|  | 4 | | 4.12 | 0.86 (0.49-1.52) | 0.618 |  |  |
| **KIV**^h^ **VP1**^g^ | |  |  |  |  | 1.03 (0.92-1.16) | 0.593 |
|  | 1 | | 6.92 | Referent |  |  |  |
|  | 2 | | 8.65 | 1.04 (0.58-1.84) | 0.905 |  |  |
|  | 3 | | 9.32 | 1.67 (0.94-2.97) | 0.086 |  |  |
|  | 4 | | 10.81 | 1.38 (0.78-2.44) | 0.273 |  |  |
| **KIV**^h^ **ST-Ag**^g^ | |  |  |  |  | 0.98 (0.88-1.09) | 0.680 |
|  | 1 | | 0.02 | Referent |  |  |  |
|  | 2 | | 0.22 | 1.03 (0.59-1.81) | 0.915 |  |  |
|  | 3 | | 3.00 | 0.69 (0.39-1.23) | 0.214 |  |  |
|  | 4 | | 4.43 | 1.10 (0.63-1.94) | 0.739 |  |  |
| **WUV**^i^ **VP1**^g^ | |  |  |  |  | 1.19 (0.94-1.49) | 0.151 |
|  | 1 | | 8.16 | Referent |  |  |  |
|  | 2 | | 8.86 | 1.37 (0.77-2.41) | 0.296 |  |  |
|  | 3 | | 9.34 | 1.15 (0.65-2.04) | 0.637 |  |  |
|  | 4 | | 10.13 | 1.49 (0.84-2.63) | 0.174 |  |  |
| **WUV**^i^ **ST-Ag**^g^ | |  |  |  |  | 1.03 (0.92-1.15) | 0.627 |
|  | 1 | | 0.02 | Referent |  |  |  |
|  | 2 | | 0.07 | 1.00 (0.57-1.76) | 0.991 |  |  |
|  | 3 | | 2.40 | 0.86 (0.49-1.51) | 0.609 |  |  |
|  | 4 | | 4.04 | 1.08 (0.61-1.89) | 0.804 |  |  |

^a^ lnMFI = natural log transformed median fluorescence intensity

^b^ Nominal (uncorrected) 95% confidence intervals

^c^ *P*-values are corrected for multiple comparisons using permutation tests.

^d^ The trend tests estimate the odds ratio for a one unit increase in natural log transformed MFI, adjusted for matched variables, ever smoking, and years of education.

^e^ MCV =Merkel cell polyomavirus

^f^ VP1 = the primary structural protein of human polyomaviruses

_g_ ST-Ag = the small T-antigen of human polyomaviruses

^h^ KIV = KI polyomavirus

^i^ WUV = WU polyomavirus
