# Supplementary material for: Absence of an association of human polyomavirus and papillomavirus infection with lung cancer in China: a nested case–control study
Source: BMC Cancer. 2016 Jun 1;16:342. doi: 10.1186/s12885-016-2381-3 (PMC4888628; doi:10.1186/s12885-016-2381-3)
Supplement: Additional file 2: — Table S2. Association between human papillomavirus (HPV) seropositivitya and incident lung cancer, adjusted for matching variables. (DOCX 26 kb) [file 12885_2016_2381_MOESM2_ESM.docx]

**Supplemental Table 2.** Association between human papillomavirus (HPV) seropositivity^a^ and incident lung cancer, adjusted for matching variables.

|  | |  | **Cases  (n=200)** | **Controls  (n=200)** |  |  |
| --- | --- | --- | --- | --- | --- | --- |
| **Antibody** | |  | **%** | **%** | **OR (95% CI**^c^**)** | ***P***^d^ |
| **HPV 16** | |  |  |  |  |  |
|  | **E6** |  | 1.6 | 1.4 | 1.18 (0.23-5.92) | 0.867 |
|  | **E7** |  | 13.7 | 12.4 | 1.12 (0.62-2.01) | 0.731 |
|  | **L1** |  | 7.7 | 5.5 | 1.40 (0.63-3.12) | 0.430 |
| **HPV 18** | |  |  |  |  |  |
|  | **E6** |  | 7.1 | 4.6 | 1.57 (0.67-3.67) | 0.350 |
|  | **E7** |  | 3.8 | 2.3 | 1.67 (0.52-5.35) | 0.440 |
|  | **L1** |  | 3.3 | 2.8 | 1.19 (0.38-3.76) | 0.770 |
| **Other high-risk HPV** | | |  |  |  |  |
|  | **31 L1** | | 12.6 | 14.7 | 0.83 (0.46-1.48) | 0.564 |
|  | **33 L1** | | 3.3 | 2.8 | 1.18 (0.37-3.73) | 0.827 |
|  | **52 L1** | | 54.6 | 52.5 | 1.09 (0.73-1.62) | 0.695 |
|  | **58 L1** | | 25.1 | 24.0 | 1.07 (0.68-1.68) | 0.850 |
| **Low-risk HPV** | | |  |  |  |  |
|  | **6 L1** | | 55.7 | 53.9 | 1.08 (0.73-1.60) | 0.736 |
|  | **11 L1** | | 35.5 | 28.6 | 1.38 (0.90-2.10) | 0.146 |

^a^ Seropositivity defined as >200 MFI (median fluorescence intensity)

^b^ The trend tests estimate the odds ratio for a one unit increase in natural log transformed MFI, adjusted for matched variables.

^c^ Nominal (uncorrected) 95% confidence intervals

^d^ *P*-values are corrected for multiple comparisons using permutation tests.
