# Supplementary material for: Absence of an association of human polyomavirus and papillomavirus infection with lung cancer in China: a nested case–control study
Source: BMC Cancer. 2016 Jun 1;16:342. doi: 10.1186/s12885-016-2381-3 (PMC4888628; doi:10.1186/s12885-016-2381-3)
Supplement: Additional file 3: — Table S3. Association between human papillomavirus (HPV) seropositivitya and incident lung cancer, adjusted for matching variables, ever smoking, and years of education. (DOCX 14 kb) [file 12885_2016_2381_MOESM3_ESM.docx]

**Supplemental Table 3.** Association between human papillomavirus (HPV) seropositivity^a^ and incident lung cancer, adjusted for matching variables, ever smoking, and years of education.

|  | |  | **Cases  (n=200)** | **Controls  (n=200)** |  |  | **Trend Test**^b^ | |
| --- | --- | --- | --- | --- | --- | --- | --- | --- |
| **Antibody** | |  | **%** | **%** | **OR (95% CI**^c^**)** | ***P***^d^ | **OR (95% CI**^c^**)** | ***P***^d^ |
| **HPV 16** | |  |  |  |  |  |  |  |
|  | **E6** |  | 0.5 | 1.4 | 0.40 (0.04-4.05) | 0.473 | 1.16 (0.95-1.41) | 0.138 |
|  | **E7** |  | 9.3 | 7.8 | 1.21 (0.59-2.47) | 0.623 | 1.05 (0.97-1.14) | 0.249 |
|  | **L1** |  | 4.4 | 3.7 | 1.25 (0.45-3.45) | 0.722 | 1.05 (0.95-1.16) | 0.367 |
| **HPV 18** | |  |  |  |  |  |  |  |
|  | **E6** |  | 1.6 | 1.4 | 1.13 (0.22-5.70) | 0.918 | 1.02 (0.92-1.13) | 0.665 |
|  | **E7** |  | 1.6 | 1.4 | 1.21 (0.23-6.34) | 0.817 | 1.07 (0.93-1.22) | 0.356 |
|  | **L1** |  | 0.5 | 1.8 | 0.29 (0.03-2.64) | 0.224 | 1.00 (0.90-1.11) | 0.980 |
| **Other high-risk HPV** | | | |  |  |  |  |  |
|  | **31 L1** | | 6.6 | 8.8 | 0.68 (0.32-1.46) | 0.339 | 1.00 (0.93-1.09) | 0.908 |
|  | **33 L1** | | 0.5 | 0.5 | 1.08 (0.07-17.71) | 0.975 | 1.03 (0.93-1.14) | 0.598 |
|  | **52 L1** | | 34.4 | 33.6 | 1.07 (0.70-1.63) | 0.769 | 1.00 (0.90-1.11) | 0.968 |
|  | **58 L1** | | 13.7 | 12.0 | 1.11 (0.61-2.01) | 0.756 | 1.03 (0.95-1.12) | 0.481 |
| **Low-risk HPV** | | | |  |  |  |  |  |
|  | **6 L1** | | 45.4 | 38.2 | 1.40 (0.93-2.10) | 0.117 | 1.03 (0.95-1.11) | 0.475 |
|  | **11 L1** | | 20.2 | 13.8 | 1.52 (0.89-2.59) | 0.131 | 1.05 (0.96-1.14) | 0.261 |

^a^ Seropositivity defined as >400 MFI (median fluorescence intensity)

^b^ The trend tests estimate the odds ratio for a one unit increase in natural log transformed MFI, adjusted for matched variables, ever smoking, and years of education.

^c^ Nominal (uncorrected) 95% confidence intervals

^d^ *P*-values are corrected for multiple comparisons using permutation tests.
